# Protective effects of ethyl pyruvate in cisplatin-induced nephrotoxicity

**DOI:** 10.1080/13102818.2014.942489

**Published:** 2014-10-27

**Authors:** Ilker Kelle, Hasan Akkoc, Selcuk Tunik, Yusuf Nergiz, Meral Erdinc, Levent Erdinc

**Affiliations:** ^a^ Department of Pharmacology, School of Medicine, Dicle University, Diyarbakir, Turkey; ^b^ Department of Histology and Embryology, School of Medicine, Dicle University, Diyarbakir, Turkey; ^c^ Department of Biochemistry, School of Medicine, Dicle University, Diyarbakir, Turkey

**Keywords:** cisplatin, nephrotoxicity, ethyl pyruvate, total oxidant status (TOS), total antioxidant status (TAS), oxidative stress index (OSI), rat

## Abstract

This study was performed to investigate the effect of ethyl pyruvate on changes in renal functions and oxidative stress related renal injury caused by cisplatin (cis-dichlorodiammine platinum-II; CDDP).

Male Wistar albino rats were divided into four groups (*n* = 8): (1) control group (1 ml Ringer's lactate solution i.p.); (2) ethyl pyruvate (EP) group (50 mg/kg Ringer's EP solution (REPS) i.p.); (3) cisplatin group (a single dose of cisplatin (5 mg/kg, i.p.); and (4) cisplatin + EP group (a single dose of cisplatin (5 mg/kg, i.p.) + REPS 50 mg/kg/day, i.p.) for five days. At the sixth day, kidneys of rats were mounted to a Langendorff apparatus. Renal perfusion pressures were recorded. Blood samples were taken for serum urea, creatinine, total oxidant status (TOS), total antioxidant status (TAS) and oxidative stres index (OSI) evaluations. Kidney tissues were obtained for malondialdehyde (MDA) analyses and histopathological examination.

Perfusion pressures, serum urea, creatinine, TOS, OSI and tissue MDA levels were found significantly higher, whereas TAS was notably lower in cisplatin group. Histopathological examination showed apparent renal paranchymal injury in cisplatin group. In cisplatin + REPS group, perfusion pressures, serum urea, creatinine and tissue MDA levels were decreased. Moreover, EP co-administration provided less inflammatory cell infiltration, tubular dilatation, whereas TOS, TAS and OSI improved significantly versus cisplatin group.

These findings show that EP has protective effects against cisplatin nephrotoxicity.

## Introductıon

Cisplatin, is one of the most effective antineoplastic drugs used in treatment of various solid tumours such as bladder, testicular and ovarian carcinomas.[1] However, irreversible renal damage induced by the agent is the main problem that occurs during clinical applications.[2] Although the exact mechanism is not clear, various studies indicate that oxidative stress plays an important role in the process and cisplatin exerts its nephrotoxic effect by generation of free radicals which mediate lipid peroxidation in kidneys.[1,3]

Kidney represents a main control system maintaining body homeostasis. The plasma concentration of urea and creatinine are indicators of renal function. These parameters are also considered as biomarkers for kidney disease. In previous studies, impairment of renal functions due to cisplatin has been reported.[1,2,4] The pathogenesis mechanisms of cisplatin-induced renal dysfunction are considered to be: reduced renal blood flow associated with increased renal vascular resistance; marked concentration defect; and proximal tubular damage.[1,5] Morphologically, the kidney damage related to cisplatin occurs particularly in the terminal portion of the proximal tubule.[6,7]

Despite of preventive precautions, irreversible renal damage occurs in about one-third of patients under cisplatin treatment. Therefore, approaches for preventing the toxicity of cisplatin are of clinical interest.[6] Thus several free radical scavengers or antioxidants have been tested in order to avoid or diminish the nephrotoxicity of cisplatin.[4,8,9]

Currently, among these agents a small pyruvate molecule, known as a key intermediate in cellular metabolism, is also considered as an effective reactive oxygen species (ROS) scavenger.[10,11] While protective effects of pyruvate have been announced in various studies,[12] in which participation of oxidative stress is suspected, the utility of pyruvate as a therapeutic agent is limited because of its poor stability in solutions. In order to overcome, this disadvantage its lipophylic ethyl esther form, ethyl pyruvate, is formulated.[13,14]

Recently, numerous studies demonstrated the positive effects of ethyl pyruvate (EP) against ischemia-reperfusion (I/R) injury,[15,16] and hemorrhagic,[17] or endotoxemic shock,[18] in various experimental models. More recently, a phase II clinical trial was conducted to assess the safety and protective potential of EP in cardiopulmonary by pass.[19]

Up to now, the derivative form of pyruvate (i.e. EP) has not yet tested in cisplatin related nephropathy. The present study aimed to investigate the potential protective effects of EP on a rat model of kidney injury induced by cisplatin.

## Мaterials and methods

### Drugs and chemicals

Cisplatin (cis-dicholorodiammine platinum-II), EP, thiobarbituric acids were obtained from Sigma chemical co. (St. Louis, USA).

### Animals and administration

Adult male Wistar albino rats (weighing 250–300 g) obtained from Dicle University Health Sciences Research Center were used in this study. In the course of experiment, the rats were kept in a 12h dark/12h light cycle room at a temperature of 23 ± 2 °C and constant relative humidity (60%), and were maintained with standard laboratory diet and water *ad libitum*. All animals received human care in accordance with the ‘Guide for the Care and Use of Laboratory Animals’ (National Institutes of Health publication 85-23, revised 1985). The experimental protocol was approved by the Dicle University Local Committee on Animal Research Ethics.

This study was performed with the following four groups each consisted of eight rats: Group 1 – control group (rats received 1 ml Ringer's lactate solution, i.p.); Group 2 – EP group (rats received a single dose of EP 50 mg/kg for five days, i.p.); Group 3 – cisplatin group (rats received a single dose of cisplatin (5 mg/kg, i.p.); Group 4 – cisplatin + EP group (rats received a single dose of cisplatin (5 mg/kg, ip) + (EP (50 mg/kg, i.p.) for five consequtive days). The doses of cisplatin and EP was determined according to previous studies.[8,20]

EP was dissolved in Ringer's lactate solution containing 130 mmol/L Na^+^, 4.0 mmol/L K^+^, 2.7 mmol/L Ca^++^ and 109 mmol/L Cl^−^ at pH 7.0.[21]

### Isolation and perfusion of kidneys

On the sixth day, pretreated rats in all groups were anesthetized by ketamine (85 mg/kg, i.m.) - Xylazine (15 mg/kg, i.m.) combination. After the administration of 500 IU heparin i.v., laparotomy was performed via a midline incision. By dissecting from surrounding tissues, one of the kidneys was removed. After cannulation of renal artery, the kidney was isolated and taken away from the abdomen and mounted on a Langendorff apparatus (MAY LS06 Ankara, Turkey). Isolated kidneys were perfused with warmed (37 °C) and aerated (5% CO_2_ in O_2_) Krebs–Henseleit solution. The composition of Krebs’ solution used was as follows (mM): NaCI 112; KCI 5; CaCI_2_ 2.5; NaHCO_3_ 25; MgCI_2_ 0.5; NaH_2_PO_4_ 1; and D-glucose 11.5. Perfusion pressure (PP) was continuously recorded on MP30 software (Biopac systems Inc., Santa Barbara, CA, USA). Kidneys were perfused with Krebs’ solution for approximately 60 minutes. After the equilibration period PP of isolated kidneys was measured and expressed as mmHg.

### Malondialdehyde analysis

Malondialdehyde (MDA) levels in the kidney tissues were determined by using the method described by Ohkawa.[22] For this purpose, a UV-1205 Shimadzu spectrophotometer was used to determine absorbance at 532 nm. The results were expressed as nmol of MDA/g tissue.

### Total antioxidant status, total oxidant status and oxidative stress index

Total oxidant status (TOS) and total antioxidant status (TAS) were measured in supernatant fraction of homogenates and serum samples (a commercially available Rel Assay Diagnostic kits with an autoanalyser (Architect c16000). TOS results were expressed in terms of micromolar hydrogen peroxide equivalent per liter (mmol H_2_O_2_ equivalent/L),[23] and TAS results were expressed as mmol Trolox equivalent/L.[24] The ratio percentage of the TOS to the TAS potential gave the oxidative stress index (OSI), an indicator of the degree of oxidative stres.[25]

### Histopathological examination

The kidneys were fixed in 10% neutral buffered formalin solution, processed for embedding in paraffin by routine protocols, and 5 μm thick sections were then cut by microtome. The sections were stained with Hematoxylin–Eosin by using a routine protocol and examined with a Nikon Eclipse 80i photomicroscope. The pathological findings of examination by using light microscopy were scored as follows: 0 (no observed changes); 1 (mild changes); 2 (moderate changes); or 3 (severe changes) and were assessed in a blinded manner.

### Statistical analysis

Statistical analysis was conducted by using the Statistical Package for the Social Sciences for Windows (version 11.0, Chicago, USA). TOS, TAS and OSI results were expressed as means ± standard deviation, and Kruskal–Wallis test was used for analysis. In the light of significant results, the Mann–Whitney *U-*test was used for comparisons of differences between two independent groups. Histopathological results were expressed as median values and analysed by Kruskal–Wallis test. A *p*-value < 0.05 was considered statistically significant.

## Results and discussion

The present study establishes that cisplatin causes a typical nephrotoxic effect which is characterized by increasing renal PP and by widespread necrosis and dilatation in terminal portion (S_3_ segment) of proximal tubules and by elevation of serum urea, creatinine, TOS, OSI and renal tissue MDA levels.

Anticancer agents which are commonly used against various types of cancer usually cause an impairment in physiological homeostasis during treatment. Side effects can arise that are induced in non-tumor cells especially by formation of free radicals and oxidant injury.[12] Thus, cisplatin therapy is limited by its nephrotoxicity which is related to oxidative stress.[3] Consequently, there is a great interest in expanding the clinical utility of cisplatin by introduction of new improved agents which decrease its toxicity. For this purpose, combination of various agents with cisplatin has been reported. Miscellaneous antioxidant compounds [26] have all been shown to reduce nephrotoxicity induced by cisplatin in experimental models. In recent studies, among these agents pyruvate, a key intermediate in anaerobic and oxidative metabolism of glucose, has been reported to have a unique feature like scavenging of ROS.[27] Following the acknowledgement of pyruvate as a free radical scavenger, numerous investigations in which oxidative stress was thought to be involved were performed with pyruvate.[10,28] Nevertheless, the utility of pyruvate as a therapeutic option has been limited by its poor solubility in solution. Instead, EP, a derivative form of pyruvate has been formulated in a Ca^2+^- and K^+^-containing solution named Ringer's ethyl pyruvate solution (REPS).[13] In time, REPS was tested for either antiinflammatory or antioxidant activity in experimental models including extrahepatic cholestasis,[29] bacterial translocation after thermal injury,[30] I/R injury,[31] and off-pump coronary bypass.[32] In all these studies, REPS has been shown to be an effective antiinflammatory and antioxidant agent. The mechanism underlying the protection constituted by EP is primarily dependant on its ROS scavenging effect.[16,29]

Serum urea and creatinine levels and the PP values of the different groups are shown in Table 1. In this study, as previously demonstrated,[1] cisplatin caused a significant increase in levels of both urea and creatinine and PP indicating the impairment of renal haemodynamics (Table 1). On the other hand, EP administration to the rats in the presence of cisplatin possessed a significant decrease in serum urea and creatinine levels as well as in renal PP as compared to the cisplatin group (Table 1). These data indicate that a significant protection of renal functions in relation to injuries caused by side effects of cisplatin was constituted by EP.

**Table 1.** Levels of MDA, urea, creatinine, perfusion pressures (PP), TOS, TAS and the OSI among the different groups.

Group	Control (*n* = 8)	EP (*n* = 8)	CIS (*n* = 8)	CIS+EP (*n* = 8)
MDA* (nmol/gram tissue)	44.5 ± 9.05	53.3 ± 8.88	143.5 ± 19.6^a^	88.6 ± 9.61^c^
Urea* (mg/dl)	38.9 ± 4.49	41.5 ± 2.30	107.2 ± 14.1^a^	58.9 ± 11.3^c^
Creatinine* (mg/dl)	0.49 ± 0.11	0.54 ± 0.10	1.76 ± 0.34^a^	0.78 ± 0.13^c^
PP* (mmHg)	56.9 ± 11.9	53.6 ± 12.4	155.4 ± 21.9^a^	69.5 ± 16.3^c^
TOS* (mmol H_2_O_2_/L)	2.97 ± 1.11	3.30 ± 0.71	5.85 ± 0.90^a^	4.08 ± 1.19^b^
TAS* (mmol Trolox/L)	0.53 ± 0.06	0.61 ± 0.02	0.39 ± 0.04^a^	0.61 ± 0.11^c^
OSI*	5.47 ± 1.69	5.66 ± 1.40	14.99 ± 3.42^a^	6.72 ± 1.99^c^

Note: EP: ethyl pyruvate treated group, CIS: cisplatin treated group, CIS+EP: cisplatin+ethyl pyruvate treated group. MDA: malondialdehyde, TOS: total oxidant status, TAS: total antioxidant status and OSI: oxidative stress index.

*p < 0.05 for Kruskal–Wallis test.

^a^ *p* < 0.01 compared to control and EP groups; ^b^ *p* < 0.05 compared to CIS group; ^c^ *p* < 0.01 compared to CIS group.

It has been demonstrated in a rat model of thermal injury[30] that both MDA and myeloperoxidase (MPO) levels were decreased by EP. MPO activity, known as the index of infiltration of polimorphonuclear neutrophils, and polimorphonuclear neutrophils are a potential source of ROS and have a crucial role in improvement of oxidative tissue injury. EP acts not only as an ROS scavenger, but also as an antiinflammatory agent; for instance, decreasing the MPO activity,[33] it is capable of preventing the development of oxidative tissue injury as well. It has been demonstrated that cisplatin accumulates by renal tubular cells and reaches its higher concentrations in the proximal tubular cells and outher medullae particularly in the S_3_ segment,[34] and cisplatin causes loss of tubular epithelial cells by necrosis and apoptosis along with inflammatory cell infiltration. Higher numbers of inflammatory cells which can augment the cytotoxic effect of ROS were also reported to be seen in renal slices in kidneys of cisplatin treated rats.[6].

**Figure 1.**
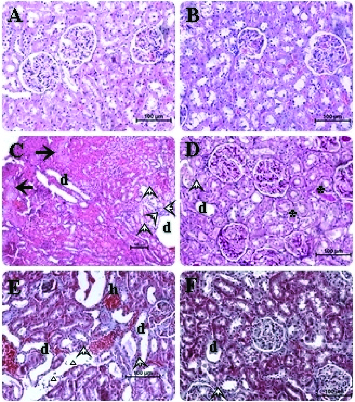
Photomicrographs of hematoxylin and eosin stained sections of kidney of rats (kidney histology magnification 200×). Typical features of normal histological appearence of the corticomedullary region of rat kidney sections are observed in control and EP administered groups (A, B). In kidney sections of cisplatin pretreated rats, marked changes were observed in tubulus and glomerule structures. In some corticomedullary regions focal tubular necrosis (big solid arrows), haemorrhagia (small letter h) and dilatation (small letter d) as well as inflammatory cell infiltration (small hollow triangle) in the intersititium can be seen. Furthermore, necrotic cell debris and vacuolization (arrow heads) in tubulus epithelium and protein casts in tubular lumina are evident (C, E). The sections obtained from the cisplatin + EP group are found almost similar to that of those in the control group. Rare inflammatory cell infiltration, minimal (asteriks symbols) tubular dilatation and vacuolization images can be seen. It is considered that the renal injury induced by cisplatin is prevented to a great extent by EP co-administration (D, F).

The histopathological examinations are demonstrated in Figure 1 and the score for tissue damage is presented in Table 2. In addition to inflammatory cell infiltration, we also observed widespread tubular necrosis and dilatation of the proximal tubules especially in the S_3_ segment and protein casts, necrotic cell debris in the tubular lumina of cisplatin group versus control group which are in agreement with previous studies (Figure 1(C) and 1(E)).[7,35]

**Table 2.** Histopathological findings in the different study groups.

	Control	EP	CIS	CIS + EP	p*
Tubular necrosis	0	0	3	1	*p* < 0.01*
Tubular dilatation and haemorrhagia	0	0	3	1	*p* < 0.01*
Necrotic cell debris, vacuolization	0	0	2	1	*p* < 0.05*
Protein casts	0	0	2	1	*p* < 0.05*
Inflammatory cell infiltration	0	0	2	1	*p* < 0.05*

Note: 0: no observed changes; 1: mild changes; 2: moderate changes; 3: severe changes. EP: ethyl pyruvate only treated group; CIS: cisplatin only treated group; CIS+EP: cisplatin+ethyl pyruvate treated group.

*CIS group was compared with the other groups.

Lipid peroxidation and inflammation are closely related with each other. Thus, in a rat model of hepatic ischemia/reperfusion injury, Tsung et al. [16] have recently reported that EP decreases hepatic lipid peroxidation and apoptosis partially by reducing neutrophil accumulation and lowering the level of inflammatory cytokines. In other studies, it has been shown that EP can inhibit the activation of pro-inflammatory signaling pathways like NF-*_K_*B,[32,36] and down-regulates the release of multiple pro-inflammatory proteins, such as IL-6 and TNF-α.[37] More recently, Yousef et al. [38] indicated that hydroxy radicals have a potential of activating mitogen-activated protein kinase which plays a critical role in cisplatin-induced acute renal damage and inflammation, through the generation of TNF-α. In an experimental model of cardioplegia, it has been shown that pyruvate has also a potential of scavenging hydroxyl radical.[11] Thus, Wang et al. [29] demonstrated that certain ROS scavengers are also anti-inflammatory agents. In this study, the severity of the tubular damage was significantly decreased in the EP co-administered group (Figure 1(D) and 1(F)) (Table 2). Less tubular dilatation and necrosis and inflammatory cell infiltration were also observed in the same EP + cisplatin group (Figure 1(D) and 1(F)) (Table 2). These data indicate that a significant compensation was constituted by EP against cisplatin nephrotoxicity. This compensation seems to be likely related with the potent antiimflammatory and antioxidant activity of EP.

The major ROS generated in the organism are superoxide anions and their derivatives particularly highly reactive hydroxyl radical attack nucleic acids, proteins, lipids and induce oxidation of these biomolecules which trigger lipid peroxidation.[21] Alterations in membrane structure and functions by lipid peroxidation induce cellular damage and are responsible for ROS-induced organ failure. Thiobarbituric acid substances (TBARS) are produced by lipid peroxidation and are considered as indicators of oxidative stress.[39]

MDA levels of the groups in this study are shown in Table 1. Renal tissue MDA (which is an end product of lipid peroxidation) levels were determined by us to be significantly higher in the cisplatin group as in agreement with previous studies (Table 1).[40] It indicates an increased lipid peroxidation and an oxidative tissue injury in kidneys. In the presence of EP, tissue injury caused by cisplatin was significantly limited in terms of decrease in MDA levels which was also established by data referring to the antioxidant activity of EP (Table 1).[29]

Oxidative stress causes an imbalance between the generation and removal of ROS. This may be due to an excessive production of ROS or by weakening of the antioxidant defence system which is typically observed during chemotherapy regimens, particularly with cisplatin treatment. From this point of view, in the present study, we assayed the oxidative status as TOS and TAS as well as the evaluation of OSI, which reflected the redox balance between oxidation and antioxidation.[41] Individual measurement of different oxidant molecules such as superoxide radical anion and hydrogen peroxide is not practical, and on the other hand their oxidant effects are additive. Thus, we measured TOS in serum as previously described by Erel [23] and Cikrikcioglu et al.[42] Likewise, we measured TAS, instead of individually determining antioxidant molecules, following the methods of Erel [24] and Horoz et al.[43] In recent studies, it has been reported that OSI may reflect the oxidative status more accurately than the levels of TOS or TAS alone.[44,45] Therefore, OSI can be referred as a predictive parameter in determining both cisplatin induced nephrotoxicity and protection obtained by EP in subjects under cisplatin therapy.

Mean levels of TOS, TAS and the OSI in this study are shown in Table 1. Among the cisplatin pretreated group, the levels of TOS and OSI were significantly increased as compared to control and EP-only-treated groups, while TAS levels markedly decreased as compared with the control (Table 1). On the other hand, the administration of EP after cisplatin reduced the levels of TOS and OSI, whereas TAS levels increased as compared to the cisplatin group (Table 1). These findings showed that cisplatin exposure induced an oxidative stress condition and that the rising of oxidative stress was prevented by the EP treatment.

### Study limitations

The aim of this study was to determine if protection effect against cisplatin nephrotoxicity can or cannot be obtained through EP treatment. Our findings should be considered as preliminary data concerning this issue. As an overview, it is evident that EP possesses antiinflammatory and antioxidant effects. However, the exact mechanisms through which EP exert these effects in cisplatin nephrotoxicity are yet to be determined. Researchers indicate the involvement of different antioxidant defence mechanisms for protection after various organ injuries related to oxidative stress. Therefore, the following steps in this field of research should evaluate the levels of endogenous antioxidant enzymes, pro-inflammatory proteins and signaling pathways in the presence of EP after cisplatin induced nephrotoxicity.

## Conclusions

The antioxidant activity of EP as a free radical scavenger along with its anti-inflammatory properties can explain the possibly involved mechanisms for nephroprotection obtained by the agent. As far as our knowledge, investigating the effects of EP in an experimental model of cisplatin-induced nephrotoxicity is one of the few studies that have been done in this area of research so far. Moreover, the results presented here in terms of preventing oxidative stress, ameliorating renal functions and hemodynamics under cisplatin exposure are promising. This may provide a unique therapeutic benefit in terms of clinical outcome such as an adjunct therapeutic option without any significant side effects for the subjects receiving cisplatin chemotherapy.

Further studies targeting different delivery systems and protocols for EP will improve the protection effect.
